# Emotional and Behavioral Consequences of the COVID-19 Pandemic: The Role of Health Anxiety, Intolerance of Uncertainty, and Distress (In)Tolerance

**DOI:** 10.3390/ijerph17197241

**Published:** 2020-10-03

**Authors:** Karoline S. Sauer, Stefanie M. Jungmann, Michael Witthöft

**Affiliations:** Department of Clinical Psychology, Psychotherapy, and Experimental Psychopathology, Johannes Gutenberg-University of Mainz, Wallstraße 3, 55122 Mainz, Germany; jungmann@uni-mainz.de (S.M.J.); witthoef@uni-mainz.de (M.W.)

**Keywords:** Covid-19, virus anxiety, safety behaviors, health anxiety, intolerance of uncertainty, distress tolerance

## Abstract

The COVID-19 pandemic represents a worldwide threat to mental health. To optimize the allocation of health care resources, research on specific vulnerability factors, such as health anxiety, intolerance of uncertainty, and distress (in)tolerance, and particularly their effect on the time course of SARS-CoV-2 related anxiety appears crucial for supporting high risk groups suffering from elevated mental distress during the pandemic. *N* = 887 participants (78.4% female; *M*_age_ = 38.15, *SD* = 17.04) completed an online survey in Germany (April to mid-May 2020), comprising measures of SARS-CoV-2 related anxiety, health anxiety, safety and preventive behavior, intolerance of uncertainty, and distress intolerance. Higher levels of health anxiety pre and during COVID-19 were associated with an initially intensified increase (*b* = 1.10, *p* < 0.001), but later on a more rapid dampening (*b* = −0.18, *p* < 0.001) of SARS-CoV-2 related anxiety. SARS-CoV-2 related preventive behavior was intensified by both pre (*b* = 0.06, *p* = 0.01) and during (*b* = 0.15, *p* < 0.001) COVID-19 health anxiety, while reassurance behavior only was associated with health anxiety during COVID-19 (*b* = 0.14, *p* < 0.001). Distress intolerance and intolerance of uncertainty did not moderate the relationship between health anxiety and SARS-CoV-2 related anxiety and behavior. The results suggest detrimental effects of health anxiety on the emotional and behavioral response to virus outbreaks.

## 1. Introduction

Several studies on the outbreak of the COVID-19 pandemic (SARS-CoV-2) showed the considerable psychological impact of the pandemic. They found that up to 28% of respondents in China suffer from symptoms of anxiety and up to 37% from depressive symptoms during the COVID-19 pandemic [1,2], which is consistent with findings from European countries (e.g., Italy [3], Spain [4]) and from previous epidemics/pandemics [5,6,7] (for an overview of the mental health impact of the COVID-19 pandemic see [8]).

Health anxiety, the anxiety of having or getting a severe (and potentially life-threatening) disease, which is conceptualized as a dimensional construct [9], could be a risk factor, influencing emotional and behavioral responses to pandemics. The predominant cognitive-behavioral model of hypochondriasis [10] proposes that dysfunctional schemata on health and illness triggered by acute stressors can lead via negative automatic thoughts and via cognitive biases in attention, memory, and interpretation of ambiguous health-related internal, and external information, and evaluation of health threat (combined-cognitive-bias hypothesis for health anxiety; [11]) to a (mis-) interpretation of potentially harmless body sensations as severe and as a sign of a life-threatening disease (=somatosensory amplification; [12]). The COVID-19 pandemic could be seen as an acute stressor with the potential to activate these beliefs and schemata and to promote the attribution of body sensations to a virus infection [13]. It can, thereby, trigger anxiety, health anxiety, and specific fears of COVID-19 [14].

Consistently, individuals with increased pre-existing health anxiety before virus outbreaks could be prone to this “vicious circle”, and to a heightened virus anxiety in the context of epidemics/pandemics [14]. The results from the COVID-19 pandemic, so far, confirmed the impact of health anxiety on the emotional responding towards the pandemic. Health anxiety and illness attitudes were associated with virus anxiety, concerns about SARS-CoV-2, COVID-19 related stress, depressive symptoms, (generalized) anxiety, death anxiety and the likelihood of self-isolation [13,15,16,17,18]. While, the latter mentioned studies did not specifically differentiate between health anxiety emerged due to COVID-19 and health anxiety existing “before” COVID-19, Taylor et al. [19,20] were the first to ask for “premorbid” health anxiety and found a significant correlation with COVID-19-related distress.

As (pre-existing) health anxiety could trigger virus-related concerns, it could also promote behavioral changes [14]. Two types of behavior are usually related to health anxiety [21]: Avoidance (e.g., body sensations, information on diseases, places/situations) and reassurance behavior (e.g., reassurance from doctors, Internet research). In the context of the COVID-19 pandemic, both behaviors could have detrimental effects: Reassurance via, e.g., overstressing the health care system and avoidance via, e.g., not obtaining the relevant information on virus spreading and protection [14]. In addition, both include negative reinforcement (e.g., short-term reduction of anxiety), which, maintains anxiety over the long-term. Highly health-anxious individuals may also be susceptible to maladaptive virus-specific preventive behaviors, such as extreme hoarding (e.g., of hygienic material) [14]. Studies on behavior during earlier epidemics/pandemics and the current COVID-19 pandemic mainly demonstrated that anxiety, worry, perceived virus threat and risks are predictors in applying recommended measures (e.g., hygienic measures and avoidance of crowded places), pursuing ineffective behavior in order to protect oneself (e.g., keeping the room temperature higher), and taking a vaccination into consideration [22,23,24,25,26,27]. With regard to hypochondriacal safety behavior, a link between COVID-19 related reassurance behavior (e.g., Internet research [incl. Internet research in the context of cyberchondria], body checking, seeking reassurance from doctors) and health anxiety was found [13,20,28]. Avoiding of SARS-CoV-2 related information by, e.g., not speaking or getting oneself informed about SARS-CoV-2, which is also typical for people with heightened health anxiety, has not been investigated in the context of the COVID-19 pandemic yet.

Intolerance of uncertainty and distress (in)tolerance are two further factors, possibly related to health anxiety and influencing responses to epidemics/pandemics. Intolerance of uncertainty is defined as the “individual’s dispositional incapacity to endure the aversive response triggered by the perceived absence of salient, key, or sufficient information, and sustained by the associated perception of uncertainty” [29] (p. 31). Concerning health anxiety, intolerance of uncertainty may have an impact on the level of dysfunctional schemata (e.g., schemata regarding the tolerance of uncertainty), on the (mis)interpretation of physical sensations via an enhanced arousal [30] and an interpretation bias [31], and on the use of safety and avoidance behavior to reduce insecurity and anxiety [32,33]. Some studies, indeed, have demonstrated an association between intolerance of uncertainty and health anxiety [34,35,36,37]. In relation to epidemics/pandemics, people with higher intolerance of uncertainty rated the H1N1 pandemic as more threatening, and their self-control and the control of others as lower, all together predicting virus anxiety [38,39]. In the context of the COVID-19 pandemic, results also indicate associations between intolerance of uncertainty and mental well-being [40], COVID-19 related distress [19], and depressive, generalized anxiety, and health anxiety symptoms [41,42].

Distress tolerance is defined as the ability to withstand aversive emotional states [43]. People with low distress tolerance rate distress as unendurable and highly aversive, and their own abilities to cope with negative psychological states aslowered [43]. They attempt to avoid aversive emotional states or feel “absorbed by the presence of distressing emotions” [43] (p. 84). Distress (in)tolerance is associated with psychopathology of many mental disorders (e.g., anxiety disorders [44]), and is linked with the use of avoidance behavior [32,45]. Fergus et al. [46] observed an association of intolerance of negative emotions (in combination with intolerance of uncertainty and intolerance of physical discomfort) with health anxiety. In the context of the COVID-19 pandemic, lower levels of distress tolerance were associated with a higher risk for clinically relevant levels of depression, anxiety, and symptoms of PTSD [47].

Some studies so far have revealed effects of health anxiety, distress (in)tolerance, and intolerance of uncertainty on virus-related anxiety and behavior in the context of the COVID-19 pandemic. These studies mostly lack a distinction between health anxiety before, and after virus outbreaks. In relation to hypochondriacal safety behavior, which is often used by high health anxious individuals, only reassurance behavior and its predictors have been investigated, whereas, to our knowledge, there are no studies on hypochondriacal avoidance behavior.

The present study aimed to overcome these limitations and to examine the specificity of effects of health anxiety, distress intolerance and intolerance of uncertainty by comparing anxiety and behavior in relation to SARS-CoV-2 and in relation to other severe diseases (e.g., cancer) or viruses. (1) We hypothesized that pre-existing health anxiety and health anxiety during the pandemic affect the course of: (a) SARS-CoV-2 related anxiety, and (b) SARS-CoV-2-related behavior (increasing levels of health anxiety both pre and during the pandemic intensify anxiety and behavior). (2) The association between health anxiety and SARS-CoV-2 related anxiety (a) and behavior (b) is assumed to be further intensified by increasing levels of intolerance of uncertainty and distress intolerance. (3) The effect of health anxiety on SARS-CoV-2 related anxiety (a) and behavior (b) should be especially seen in comparison with anxiety in relation to other severe diseases/viruses.

## 2. Materials and Methods

### 2.1. Participants and Procedure

Participants in the online survey (*N* = 887) were recruited via social media (e.g., Twitter), university mailing lists, and press releases (print, online). A minimum age of 18 years and an informed consent were required as eligibility criteria. As an expense allowance, participants could take part in a lottery of gift vouchers or receive a course credit (psychology students). The online survey was clicked *N* = 2009 and started *N* = 1081 times. Most dropouts (*N* = 106) were registered on the first pages of the survey. From the *N* = 887 participants, 78.4% were female, 21.2% male, and 0.5% diverse. The mean age was *M* = 38.15 years (*SD* = 17.04; range: 18–85 years). Regarding formal education, 1.8% had completed primary school or had a basic school leaving qualification, 8.9% a secondary school certificate, 4.7% an advanced technical college certificate, 34.8% a higher education entrance qualification and 49.6% a university degree. Regarding current profession, 0.1% were school students, 1.1% in professional training, 42.2% college students (of whom 67.9% psychology), 30.4% employees, 4.5% civil servants, 6.1% self-employees, 1.1% job-seekers, 10.3% pensioners, 1.1% housewives or househusbands, 1.1% in parental leave and 2.4% reported to pursue another profession (e.g., voluntary social year). 26.4% of the study participants were reported to have a physical disease, 3.9% to take an immunosuppressive medication, and 15.2% to suffer from a mental disorder. 0.5% stated that they have been infected with SARS-CoV-2 and 9.7% or 34.4% reported that someone from their closer surrounding (family/friends) or wider environment (circle of acquaintances, residents in the home town) was infected with SARS-CoV-2. 0.9% of all participants stated that they are in voluntary or enforced quarantine.

Recruitment took place between the 20th of April and 15th of May 2020 (25 days). On the 20th of April, Germany had *N* = 141,672 registered cumulated SARS-CoV-2 infections and *N* = 1,775 newly reported SARS-CoV-2 infections on that day [48]. On the 15th of May, Germany had *N* = 173,152 registered cumulated SARS-CoV-2 infections and *N* = 913 newly reported SARS-CoV-2 infections on that day [49]. During recruitment, previously set government restrictions on contacts and opening of public places/stores were gradually eased (e.g., since 20th of March 2020: Reopening of certain shops; since the 6th of May 2020: Permission of one contact beyond the same household).

The study protocol was approved by the local ethics committee of the Department of Psychology of Johannes Gutenberg-University of Mainz (2020-JGU-psychEK-S007).

### 2.2. Measures

Health anxiety was measured with the Short Health Anxiety Inventory (SHAI; [50]) consisting of 18 multiple-choice items (four choices for each item; coding 0 to 3). The items can be aggregated to two factors: “health anxiety” (14 items) and “negative consequences of illness” (4 items). In the present study, the validated German version of the SHAI [51] was adapted to assess health anxiety before, and during the outbreak of the COVID-19 pandemic (e.g., “Prior to/since the onset of the COVID-19 pandemic [in Germany before/after the end of January]: I didn’t/don’t worry (0), occasionally worried/worry (1), spent/spend much of my time worrying (2), spent/spend most of my time worrying (3) about my health.”). Internal consistencies in the present study were α = 0.89 (pre COVID-19) and α = 0.92 (during COVID-19) for the total scale, α = 0.89 (pre COVID-19) and α = 0.92 (during COVID-19) for the subscale “health anxiety” and α = 0.67 (pre COVID-19) and α = 0.71 (during COVID-19) for the subscale “negative consequences of illness”.

The German Questionnaire for assessing hypochondriacal safety behavior (QSBH; [52]) assesses hypochondriacal safety behavior and consists of 16 items rated on a 5-point Likert scale from 0 (“never”) to 4 (“very often”) with two subscales (reassurance and avoidance behavior, each 8 items). A sum score is calculated for both subscales. The QSBH has demonstrated good to excellent internal consistency, and satisfactory convergent and discriminant validity [52]. For the present study, the QSBH was adapted to measure hypochondriacal safety behavior prior (e.g., “Prior to the onset of the COVID-19 pandemic: Did you check the Internet on symptoms and potential diseases?”) and during the COVID-19 pandemic in relation to SARS-CoV-2 (incl. COVID-19) and in relation to other severe diseases (not COVID-19; e.g., cancer) (e.g., “Since the onset of the COVID-19 pandemic: Do you check the Internet on symptoms of a coronavirus infection and potential secondary diseases [e.g., COVID-19]/on symptoms or other severe diseases [not COVID-19; e.g., cancer]?”). Five questions of the QSBH were difficult to adapt to assess behavior in relation to SARS-CoV-2 (e.g., “Do you check your birthmarks on abnormalities?”); these questions were only presented once at the time point “since the COVID-19 pandemic”. Additional questions, specific to preventive behavior in pandemics, were added at both time points (pre and during COVID-19) with regard to SARS-CoV-2 and other viruses: (1) Buy toiletries, e.g., disinfectants, (2) wash your hands, (3) buy and/or use respiratory masks, (4) avoid places where you can get infected. In the present study, the internal consistencies were α_reassurance_ = 0.74 and α_avoidance_ = 0.84 pre COVID-19, α_reassurance_ = 0.78 and α_avoidance_ = 0.89 during COVID-19 regarding other severe diseases, and α_reassurance_ = 0.78 and α_avoidance_ = 0.86 during COVID-19 regarding SARS-CoV-2. Internal consistencies of the items to assess preventive behavior were α = 0.74 both pre and during COVID-19 and in relation to SARS-CoV-2 and in relation to other viruses.

To measure intolerance of uncertainty, the short German version [53] of the 27-item Intolerance of Uncertainty Scale (IUS; [54,55]), which consists of 18 items measuring three aspects of intolerance of uncertainty (“reduced ability to act due to IU”, “burden due to IU”, and “vigilance due to IU”) on a 5-point Likert scale (1 = “not at all characteristic of me”, 5 = “entirely characteristic of me”), was applied. A total score — in addition to subscale scores — can be computed by summing up all items. The German IU-18 has demonstrated good internal consistency and satisfying retest reliability and validity in a non-clinical sample [53]. The internal consistencies in the present study were α_total scale_ = 0.94, α_vigilance_ = 0.85, α_burden_ = 0.87, and α_reduced ability_ = 0.88.

The Distress Intolerance Scale [56], a 10-item questionnaire on a 5-point Likert Scale from 1 (“I don’t agree at all.”) to 5 (“I totally agree.”), measures the “perceived inability to tolerate negative somatic and emotional states” [56] (p. 641). It was developed based on a confirmatory factor analysis of the Anxiety Sensitivity Index (ASI; [57]), the Frustration Discomfort Scale (FDS; [58]), the Discomfort Intolerance Scale (DIS; [59]), and the Distress Tolerance Scale (DTS; [43]). A first analysis of the reliability and validity of the German version of the Distress Intolerance Scale [60] suggested a satisfying internal consistency and convergent validity. The internal consistency in the present study was α = 0.93.

As in a previous study on coronavirus anxiety [13], participants were additionally asked to (retrospectively) rate their anxiety (0 = “no anxiety” to 100 = “very intense anxiety”), in relation to SARS-CoV-2 and additionally in relation to other severe diseases at four time points (December 2019 [first reports of coronavirus infections in China], January 2020 [first reports of coronavirus infections in Germany], March 2020 [increasing restriction of contacts in Germany], and past week). Furthermore, a question on people’s most feared disease (“Which disease/infection do you fear most to have and/or to get at present times?”) with single selection (coronavirus infection [incl. COVID-19], cancer, cardiovascular diseases, injuries/poisoning, other infections [not SARS-CoV-2], respiratory diseases, neurological diseases, mental disorders), free description or “no disease” option was administered.

### 2.3. Statistical Analyses

Dependent (paired) sample t-tests, including bootstrap method with BCa adjustment and 5000 reiterations, were calculated to test for a change of health anxiety pre to during COVID-19 and to compare behavior in relation to SARS-CoV-2 and to other severe diseases.

Multilevel growth models with maximum likelihood estimation (MLE) were used to investigate the course of SARS-CoV-2 related anxiety and the effects of health anxiety, intolerance of uncertainty, and distress intolerance on the growth of anxiety. According to Singer and Willett [61], modeling was carried out in three stages: (1) Calculating the unconditional means model, (2) fitting unconditional growth models, and (3) calculating conditional growth models by adding health anxiety on level 1 and intolerance of uncertainty and distress intolerance on level 2 (all grand mean centered). The points of measurement were recoded so that the spacing was constant (December 2019 = 0). Decisions on selecting trajectories, including random effects of polynomials and influencing variables and choosing covariance structure, were taken on the basis of the overall fit of the multilevel model by comparing the Akaike’s Information Criterion (AIC) and the Schwarz’s Bayesian Criterion (BIC) of different models. In the results section only the best-fitting unconditional and conditional models are presented.

A repeated measures analysis of covariance (rmANCOVA) with two within-subject factors (1: disease/infection type [SARS-CoV-2 versus other severe diseases]; 2: time [December 2019, January 2020, March 2020, past week]) and centered health anxiety pre COVID-19 as a covariate was calculated to compare the effect of pre-existing health anxiety on anxiety related to SARS-CoV-2 and to other severe diseases (Greenhouse-Geisser adjustment for violations of sphericity).

(Moderator) hypotheses of health anxiety, distress intolerance, and intolerance of uncertainty on behavior during COVID-19 were analyzed with the PROCESS macro in SPSS [62], using bootstrap method with BCa adjustment, 5000 reiterations, and the heteroskedasticity-consistent standard error estimator HC4. Multicollinearity was assessed via calculating tolerance values with values smaller than 0.40 being considered as a sign of multicollinearity. Due to multicollinearity issues regarding health anxiety pre and during COVID-19, separate moderator analyses on reassurance, avoidance, and preventive behavior in relation to SARS-CoV-2 and to other severe diseases/viruses were calculated for health anxiety pre and during COVID-19 (corresponding behavior pre COVID-19 was included as a covariate). 

Due to multiple testing and in order to avoid an inflated Type I error, the alpha level was set at *p* = 0.01 for all tests.

## 3. Results

### 3.1. Course of Health Anxiety and SARS-CoV-2 Related Anxiety

Table 1 shows the statistics of all measured variables. Supplementary Table S1 provides additional correlational analyses of anxiety, health anxiety, behavior, and sociodemographic and other variables (e.g., pre-existing mental disorder) (We investigated the effects of the variables age, sex, education, pre-existing physical disease, and mental disorder on the outcomes by including the variables as covariates and/or performing subgroup analyses with sex [female and male] and age [“young”: 18-29, “middle”: 30-59, “old”: ≥ 60 years]. All previously reported results remained stable, except for the comparison of behavior in relation to SARS-CoV-2 and to other diseases/viruses [3.2; subgroup analyses are reported].). Health anxiety significantly increased (difference BCa 99% CI [2.73, 3.62]) since the outbreak of the COVID-19 pandemic (*t*(886) = |18.64|, *p* < 0.001, *d* = 0.39), which was also shown on both subscales (“health anxiety”: *M*_pre_ = 11.08, *SD*_pre_ = 5.97; *M*_during_ = 13.88, *SD*_during_ = 7.04; difference BCa 99% CI [2.42, 3.19]; *t*(886) = |19.11|, *p* < 0.001, *d* = 0.42; “negative consequences of illness”: *M*_pre_ = 2.92, *SD*_pre_ = 1.97; *M*_during_ = 3.29, *SD*_during_ = 2.18; difference BCa 99% CI [0.27, 0.47]; *t*(886) = |9.57|, *p* < 0.001, *d* = 0.18).

**Figure 1:**
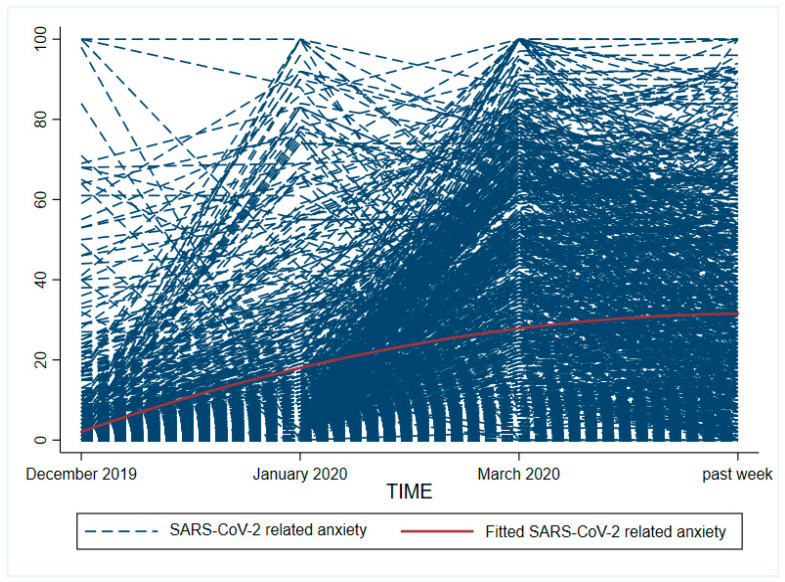
Course of anxiety in relation to SARS-CoV-2 (including COVID-19) on individual level during the COVID-19 pandemic (December 2019, January 2020 and March 2020 retrospectively assessed) (fitted trajectory of sample development added).

An unconditional growth model with a linear and quadratic slope was found to be the most parsimonious to model the development of SARS-CoV-2 related anxiety during the COVID-19 pandemic. The model revealed a significant intercept (*b*_0_ = 2.08, *SE* = 0.58, *p* < 0.001), positive linear slope parameter (*b*_1_ = 18.97, *SE* = 0.92, *p* < 0.001), and negative quadratic slope parameter (*b*_2_ = −3.05, *SE* = 0.31, *p* < 0.001). A significant linear increase of SARS-CoV-2 related anxiety until the last assessed time point (past week) was observed. This trend levels off over time, which is shown by a negative quadratic slope (Figure 1). There was a significant variance in the intercept, linear and quadratic slope across all participants (*b* = 7.28, *SE* = 0.49, *p* < 0.001), indicating variation in the initial level of SARS-CoV-2 related anxiety in December 2019 and in the linear and quadratic growth of anxiety between participants.

### 3.2. Comparison of Behavior in Relation to SARS-CoV-2 and to Other Diseases/Viruses

As some sex and age differences regarding the comparison of SARS-CoV-2 related behavior with behavior in relation to other diseases/viruses were found, results are reported separately for women and men (diverse excluded due to the low frequency) and age groups (“young”: 18–29, “middle”: 30–59, “old”: ≥60 years). As shown in Table 2, women reported more reassurance, avoidance, and preventive behavior in relation to SARS-CoV-2 than to other diseases/viruses, while men only reported more SARS-CoV-2 related reassurance and preventive behavior (no significant difference regarding avoidance behavior). More preventive and reassurance behavior in relation to SARS-CoV-2 (compared with behavior in relation to other diseases/viruses) was observed in all age groups. Divergent results were observed regarding hypochondriacal avoidance behavior (more SARS-CoV-2 related avoidance behavior in “young” and less in “old”; inconclusive regarding direction for “middle”).

### 3.3. Prediction of SARS-CoV-2 Related Anxiety during the COVID-19 Pandemic

In the conditional growth model of SARS-CoV-2 related anxiety, a significant positive fixed interaction effect of health anxiety (*b*_4_ = 1.10, *SE* = 0.14, *p* < 0.001) with the positive linear slope and a significant negative fixed interaction effect of health anxiety with the negative quadratic slope (*b*_5_ = −0.18, *SE* = 0.04, *p* < 0.001) were shown. SARS-CoV-2 related anxiety intensified with increasing levels of health anxiety over the course of the pandemic. Increasing levels of health anxiety were furthermore associated with an intensified dampening of the increase over time (interaction with the negative quadratic slope). No significant fixed and interaction effects (with health anxiety) of intolerance of uncertainty and distress intolerance on SARS-CoV-2 related anxiety were found (Table 3).

### 3.4. Comparison of the Effect of Pre-Existing Health Anxiety on Anxiety in Relation to SARS-CoV-2 and to Other Severe Diseases

**Figure 2:**
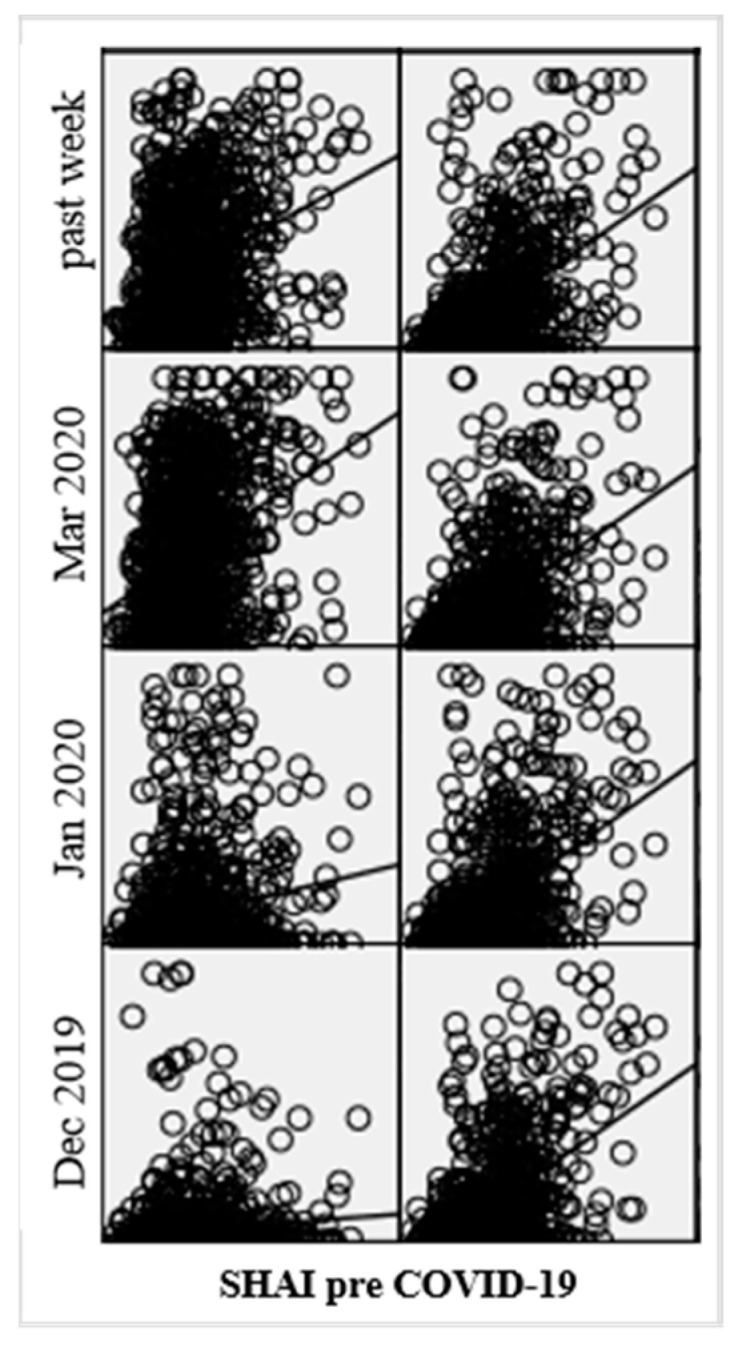
Anxiety in relation to SARS-CoV-2 (including COVID-19) (left) and in relation to other severe diseases (not COVID-19; e.g., cancer) (right) as a function of health anxiety (SHAI) pre COVID-19 over the course of the COVID-19 pandemic (December [Dec] 2019, January [Jan] 2020, and March [Mar] 2020, retrospectively assessed).

The repeated measures ANCOVA with two within-subject factors and centered health anxiety pre COVID-19 as a covariate revealed small to medium interaction effects time × health anxiety pre COVID-19 (*F*(2.29, 2024.70) = 34.65, *p* < 0.001, η_p_^2^ = 0.04), health anxiety pre COVID-19 × disease/infection type (*F*(1, 885) = 53.65, *p* < 0.001, η_p_^2^ = 0.06), and time × health anxiety pre COVID-19 × disease/infection type (*F*(2.27, 2012.40) = 44.05, *p* < 0.001, η_p_^2^ = 0.05). Figure 2 depicts anxiety in relation to SARS-CoV-2 and in relation to other severe diseases as a function of health anxiety pre COVID-19. The effect of health anxiety pre COVID-19 on anxiety related to other severe diseases seemed to be relatively constant over time, whereas the regression line of health anxiety pre on SARS-CoV-2 related anxiety got steeper until March 2020 and flattened after March 2020.

### 3.5. Prediction of SARS-CoV-2 Related Behavior in the Context of the COVID-19 Pandemic and Comparison with Behavior in Relation to Other Severe Diseases/Viruses

Table 4 and Table 5 show the moderator analyses on reassurance, avoidance and preventive behavior with regard to SARS-CoV-2 and to other severe diseases/viruses. There was only a significant positive effect of health anxiety pre COVID-19 on SARS-CoV-2 related preventive behavior (*b* = 0.06, 99% CI [0.01, 0.11], *p* = 0.005), but no effects on both reassurance and avoidance behavior (both in relation to SARS-CoV-2 and other diseases), and on preventive behavior in relation to other viruses. Health anxiety during COVID-19, on the contrary, significantly positively predicted reassurance (*b*_SARS-CoV-2_ = 0.14, 99% CI [0.10, 0.19], *p* < 0.001; *b*_diseases_ = 0.06, 99% CI [0.03, 0.09], *p* < 0.001) and preventive behavior (*b*_SARS-CoV-2_ = 0.15, 99% CI [0.11, 0.20], *p* < 0.001; *b*_viruses_ = 0.05, 99% CI [0.01, 0.09], *p* = 0.002). Concerning avoidance behavior, there was only a marginally significant, but inconclusive effect of health anxiety during COVID-19 on avoidance behavior in relation to other diseases (*b* = 0.03, 99% CI [−0.01, 0.06], *p* = 0.08).

Besides a marginally significant and inconclusive interaction effect health anxiety pre COVID-19 × intolerance of uncertainty (*b* = 0.003, 99% CI [−0.001, 0.01], *p* = 0.09) based on a non-significant predictor (*p* = 0.96), none of the assumed moderator effects of distress intolerance and intolerance of uncertainty proved to be significant. Conditional positive effects of distress intolerance on avoidance behavior in all moderator analyses (*b*_SARS-CoV-2_ = 0.07, 99% CI [0.02, 0.12], *p* = 0.001; *b*_diseases, HA pre_ = 0.04, 99% CI [0.002, 0.08], *p* = 0.01; *b*_diseases, HA during_ = 0.03, 99% CI [0.002, 0.07], *p* = 0.01) and inconsistent effects of distress intolerance on reassurance behavior in the moderator analyses with health anxiety pre COVID-19 (*b*_SARS-CoV-2_ = 0.04, 99% CI [−0.01, 0.08], *p* = 0.03; *b*_diseases_ = 0.03, 99% CI [0.01, 0.06], *p* = 0.003) and partly with health anxiety during COVID-19 (*b*_diseases_ = 0.02, 99% CI [−0.004, 0.05], *p* = 0.04) could be observed. Regarding intolerance of uncertainty, we only found a marginally significant and inconsistent conditional effect on reassurance behavior in relation to other severe diseases (moderator analysis with health anxiety during COVID-19: *b* = −0.01, 99% CI [−0.03, 0.005], *p* = 0.07). In all moderator analyses, pre COVID-19 behavior proofed to be a significant positive predictor of during COVID-19 behavior (especially in relation to other diseases/viruses).

## 4. Discussion

The present study investigated the course of SARS-CoV-2-related anxiety and behaviors, and the contributing effects of health anxiety, distress (in)tolerance, and intolerance of uncertainty in the context of the COVID-19 pandemic in Germany.

While health anxiety intensified during the COVID-19 pandemic as expected, SARS-CoV-2 related anxiety increased but — contrary to our assumptions — leveled off over the course of the COVID-19 pandemic (especially in April and May 2020). This result can be explained by the timing of the survey. Germany went through the beginning and “first peak” of the COVID-19 pandemic (with regard to daily reported infections) in January and March 2020, leading to several measures to reduce virus spreading. In April and May 2020, the rate of infection has decreased considerably (e.g., seven-day-reproduction rate *R* = 1.99 [15th of March 2020] vs. *R* = 0.84 [20th of April 2020] vs. *R* = 0.91 [15th of May 2020]; [63]) and some government restrictions have been loosened. A changed “climate” regarding the COVID-19 pandemic, more security/knowledge about the pandemic or a “habituation-like effect” to virus threat could have changed risk assessment regarding SARS-CoV-2 and led to a dampening of anxiety after March 2020. The results from previous epidemics/pandemics confirmed that responses to virus outbreaks are variable and can decrease over the course of epidemics/pandemics [64,65].

People seemed to follow recommendations from German officialities on preventive behavior and more frequently used it in order to protect themselves against SARS-CoV-2 than any other infection, which was seen in all age groups and both sexes. Hygienic measures (e.g., washing hands) were, as in previous studies (e.g., [66]), most frequently reported. In relation to hypochondriacal safety behavior, the results on the subgroup level were less conclusive, but still indicated a continuously high need for information on SARS-CoV-2 in the investigated sample.

Previous studies have highlighted health anxiety as a potential risk factor for heightened virus anxiety [13,15,16,17,18,19,20], but partly lacked a differentiation between health anxiety pre and during COVID-19. In the present study, we confirmed the role of health anxiety in SARS-CoV-2 related anxiety and additionally showed that both pre-existing (pre) (retrospectively assessed) and emerging (during) health anxiety intensified the linear increase of SARS-CoV-2 related anxiety during the COVID-19 pandemic. We furthermore found that health anxiety was also significantly associated with the negative quadratic slope of SARS-CoV-2 related anxiety, this time indicating a stronger and/or faster dampening of the increase of SARS-CoV-2 related anxiety over the course of the COVID-19 pandemic with increasing levels of health anxiety. The first finding is consistent with our assumptions and the results of previous studies (e.g., [13]); the latter finding, on the contrary, is unexpected, as one could assume that the dampening of SARS-CoV-2 related anxiety should not be intensified or fastened but rather diminished or slowed down with increasing levels of health anxiety. One interpretation of this finding could be that people with heightened health anxiety are still most concerned about the disease they feared most pre COVID-19 (e.g., cancer, cardiovascular diseases), and therefore, rapidly switched the subject of their health worries back to the pre COVID-19 most feared disease, when SARS-CoV-2 was not that present in the public eye anymore (April/May 2020). However, when virus anxiety was quite “virulent” in the general population (March 2020; “first peak” phase), the pandemic seemed to trigger specific schemata and automatic thoughts on health/illness in high health anxious people and led to an intensification of SARS-CoV-2 related anxiety. These interpretations are further underpinned by the finding, that SARS-CoV-2 related anxiety was especially strongly associated with pre COVID-19 health anxiety in the “first peak” (March 2020) and the “later” (April/May 2020) phase of the COVID-19 pandemic, whereas anxiety in relation to other severe diseases (e.g., cancer) was quite constantly affected by pre-existing health anxiety over the course of the COVID-19 pandemic. Another indication for the assumption of a “switching” of health worries (from SARS-CoV-2 to other severe diseases) in high health anxious people could be seen in the finding, that at the time of assessment (April/May 2020) almost an equal amount of people (about 30%) feared most an infection with SARS-CoV-2 and any other disease/infection. SARS-CoV-2 (incl. COVID-19), therefore, was not the predominant feared disease in all assessed people. Nevertheless, the findings still indicate the need to support possible risk groups (e.g., high health anxious individuals) during epidemics/pandemics. It furthermore underlines the relevance of conducting more research on long-term effects and coping of vulnerable groups in epidemics/pandemics, and even after the subsiding of epidemics/pandemics.

With regard to the role of health anxiety in SARS-CoV-2 related behavior, we found that health anxiety during COVID-19 intensified SARS-CoV-2 related reassurance and preventive behavior as expected, while no significant effect on SARS-CoV-2 related avoidance behavior was observed. This pattern was also observed with regard to other diseases/viruses; however, the effects of emerging health anxiety on reassurance and preventive behavior seemed to be more intensive for SARS-CoV-2 than for other diseases/viruses. These results are consistent with previously shown associations between health anxiety and COVID-19 related reassurance [13,20,28] and preventive behavior [15,22,23].

Contrary to our assumptions, we only found an increasing effect of pre-existing health anxiety (pre COVID-19 health anxiety) on SARS-CoV-2 related preventive behavior, but no associations between pre-existing health anxiety and SARS-CoV-2 related avoidance and reassurance behavior. The “non-significance” concerning avoidance behavior could be again interpreted as a sign of a high need for information regarding SARS-CoV-2 (possibly also due to the perceived novelty of the virus/pandemic situation) in “all levels” of pre-existing health anxiety. The latter result regarding reassurance behavior, on the contrary, is inconsistent with the previous shown medium correlation between SARS-CoV-2 related reassurance seeking behavior (e.g., body-checking, doctor visits) and pre COVID-19 health anxiety [20]. It could indicate, that emerging health worries are more relevant for the wish to seek reassurance regarding SARS-CoV-2 than the intensity of pre-existing health anxiety. Furthermore, one could interpret that reassurance (and avoidance) in the context of the COVID-19 pandemic (and possibly also other epidemics/pandemics) is less specific for people with “previously” heightened high health anxiety, but can be seen in many people with “newly” triggered/intensified health worries.

Still, most predictive of behavior since the outbreak of the COVID-19 was pre COVID-19 behavior. Behavior seems to stay relatively constant over time. There is a lack of comparable studies to confirm this, but hypochondriasis, which is characterized by reassurance and avoidance behavior, is known to be temporally quite stable [67]. Consistently, it is not surprising that more frequent previous behavior especially led to a stronger increase in behavior in relation to other diseases/viruses during COVID-19, and seemed to affect SARS-CoV-2 related behavior to a lesser extent. In line with this finding, only a small part of variance of SARS-CoV-2 related behavior (in contrast to behavior in relation to other diseases/viruses) could be explained by the included variables (health anxiety, distress intolerance, intolerance of uncertainty), which suggests the importance of other vulnerability factors not investigated in the present study.

Contrary to our hypotheses, no “interpretable” moderating effect of distress intolerance and intolerance of uncertainty on the association between health anxiety (pre and during COVID-19) and SARS-CoV-2 related anxiety and behavior was shown. In relation to conditional effects, an intensifying effect of distress intolerance on SARS-CoV-2 related avoidance behavior was found, which is consistent with other results on the association of avoidance behavior and distress (in)tolerance [32,45]. Although we observed conditional (marginally) significant effects of distress intolerance and intolerance of uncertainty on reassurance behavior (for intolerance of uncertainty only an association with reassurance behavior in relation to other diseases was shown), the direction of these effects was partly inconclusive which complicates interpretation. Either way, the previously indicated crucial role of distress intolerance and intolerance of uncertainty in the emotional and behavioral response to the COVID-19 pandemic could not be confirmed in the present study. As the conceptualization of distress (in)tolerance as distinctive from other constructs still remains inconclusive [68,69], distress could therefore be defined as a broader construct also comprising uncertainty as a possible cause of distress. This could lead to a strong interrelation of both constructs and could have reduced separate predictive value in the present study.

In summary, the present study emphasizes the need to support people with vulnerability factors, such as heightened health anxiety, in epidemics/pandemics, as they seem to be prone to heightened distress and intensified behavioral responses to epidemics/pandemics. It underlines the importance of conducting longitudinal studies to enable conclusions on the long-time development of mental health during the course of epidemics/pandemics and emphasizes the need for research on behavior in epidemics/pandemics. Although, detrimental effects of health anxiety (both pre-existing and emerging) on behavior in the context of the COVID-19 pandemic could be seen, these effects were less consistent and conclusive than expected, indicating differential behavioral responses to the COVID-19 pandemic.

Some limitations should be addressed. First, the survey sample cannot be considered as representative of the German general population as it differs in age (younger), sex (high proportion of female people), education (higher), and profession (higher). Especially the high proportion of college students (about 40%) and the high proportion of people with female sex (about 80%) could skew the results (e.g., intensify mental distress). Second, some outcomes (anxiety, health anxiety, and behavior) were retrospectively assessed, which makes the results vulnerable to a recall bias. Furthermore, they were adapted (SHAI, QSBH) to allow a separate assessment of different time points and/or contents. This could have reduced the quality of the outcome measures. However, internal consistency of the adapted measures was still satisfying. The adding of items to assess preventive behavior and the use of a non-validated measure of anxiety could limit comparability and generalization of the corresponding results. Future studies should incorporate newly published instruments to assess SARS-CoV-2 related anxiety (e.g., COVID stress scales; [20]). Measures assessing specific behavior in pandemics/epidemics and differentiating regarding (dys)functionality of behavior should be developed. The present study lacks a differentiation regarding adaptivity and functionality of behavior.

## 5. Conclusions

Overall, the present study indicates that pre-existing health anxiety and health worries, which emerge during epidemics/pandemics influence the affective and behavioral responses to virus outbreaks. Increasing levels of health anxiety pre and during COVID-19 were associated with a stronger increase and—at the same time—a stronger/faster dampening of SARS-CoV-2 related anxiety (in the “later” phase [April/May 2020]) over the course of the COVID-19 pandemic in Germany. The effect of pre-existing health worries was especially strong in the investigated “first peak” (March 2020) and “later” (April to May 2020) phase of the COVID-19 pandemic, while anxiety with regard to other severe diseases (e.g., cancer) was quite constantly affected by pre-existing health anxiety. Health anxiety during COVID-19 was associated with an increase of hypochondriacal reassurance and preventive behavior, whereas pre-existing health anxiety only intensified preventive behavior specifically related to SARS-CoV-2. Intolerance of uncertainty and distress intolerance did not moderate the association between pre-existing health anxiety and SARS-CoV-2 related anxiety or behavior. Nevertheless, an increasing effect of distress intolerance on avoidance behavior was shown. The study implies that more research on behavioral responses to virus outbreaks as well as more longitudinal studies investigating the course of emotional and behavioral responses during epidemics/pandemics are needed. The results furthermore highlight a potential risk group (with heightened health anxiety) in need for more support during epidemics/pandemics. Providers in the healthcare system should be sensitive to the mental health burden of this risk group and should ensure easy access to qualified information and support measures, which at best should be individually adapted and gradually made available on the basis of COVID-19 related stress (e.g., from Internet information to low-threshold counseling and psychotherapy). In particular, a transparent information policy which keeps the balance between pointing out risks, promoting functional behavior, and avoiding sensational reporting styles appears crucial. Different wide-scope supportive approaches, like tele counselling, appear promising, but still lack a rigorous empirical evaluation.

## Figures and Tables

**Table 1:** Descriptive statistics of applied questionnaires and ratings.

	*M*	*SD*	*N*	*%*
Anxiety related to SARS-CoV-2 (0–100)				
December 2019 ^a^	4.44	11.97		
January 2020 ^a^	10.93	19.37		
March 2020 ^a^	34.90	27.97		
past week	29.20	25.37		
Anxiety related to other severe diseases (0–100)				
December 2019 ^a^	12.21	18.86		
January 2020 ^a^	13.19	20.16		
March 2020 ^a^	13.24	19.70		
past week	13.22	19.81		
Health anxiety (SHAI) pre COVID-19 ^b^				
total (0–54)	14.00	7.13		
subscale “health anxiety” (0–42)	11.08	5.97		
subscale “negative consequences of illness” (0–12)	2.92	1.97		
Health anxiety (SHAI) during COVID-19				
total (0–54)	17.16	8.45		
subscale “health anxiety” (0–42)	13.88	7.04		
subscale “negative consequences of illness” (0–12)	3.29	2.18		
Hypochondriacal safety behavior (QSBH) pre COVID-19 ^c^				
subscale “reassurance” (original version) (0–32)	9.88	4.92		
subscale “avoidance” (original version) (0–32)	11.26	6.77		
subscale “reassurance” (shortened version) (0–20)	6.33	3.39		
subscale “avoidance” (shortened version) (0–20)	4.56	4.29		
Hypochondriacal safety behavior (QSBH) during COVID-19				
subscale “reassurance”—SARS-CoV-2 (0–20)	6.53	3.75		
subscale “reassurance”—other severe diseases (0–20)	5.90	3.53		
subscale “avoidance”—SARS-CoV-2 (0–20)	4.36	4.20		
subscale “avoidance”—other severe diseases (0–20)	4.17	4.49		
Preventive behavior pre COVID-19 ^d^				
total (0–16)	4.70	3.12		
Buying or using toiletries (e.g., disinfectants)	1.21	1.08		
Washing hands	2.33	1.10		
Buying or using respiratory masks	0.30	0.85		
Avoiding places	0.85	1.11		
Preventive behavior during COVID-19				
total—SARS-CoV-2 (0–16)	10.75	3.50		
total—other infections (0–16)	5.24	3.45		
Buying or using toiletries (e.g., disinfectants)—SARS-CoV-2	2.26	1.23		
Buying or using toiletries (e.g., disinfectants)—other infections	1.36	1.21		
Washing hands—SARS-CoV-2	3.35	0.84		
Washing hands—other infections	2.35	1.19		
Buying or using respiratory masks—SARS-CoV-2	2.48	1.29		
Buying or using respiratory masks—other infections	0.41	0.92		
Avoiding places—SARS-CoV-2	2.66	1.25		
Avoiding places—other infections	1.11	1.25		
Most feared disease at the moment				
Infection with SARS-CoV-2 (incl. COVID-19)			292	32.90
Cancer			107	12.10
Mental disorders			93	10.50
Cardiovascular diseases			63	7.10
Respiratory diseases			15	1.70
Neurological diseases			14	1.60
Injuries/poisoning			7	0.80
Other infections			3	0.30
Others			22	2.50
No disease			271	30.60
Intolerance of uncertainty (IU-18)				
total (18–90)	47.39	14.82		
subscale “reduced ability to act due to IU” (6–30)	14.13	5.71		
subscale “burden due to IU” (6–30)	16.64	5.61		
subscale “vigilance due to IU” (6–30)	16.62	5.37		
Distress intolerance (Distress intolerance scale)				
total (10–50)	24.63	9.34		

SHAI = Short Health Anxiety Inventory. QSBH = Questionnaire for assessing safety behavior in patients with hypochondriasis. IU-18 = Intolerance of Uncertainty Scale—short version. ^a^ Anxiety related to SARS-CoV-2 and other severe diseases in December 2019, January 2020 and March 2020 were retrospectively assessed. ^b^ Health anxiety (SHAI) pre COVID-19 was retrospectively assessed. ^c^ Hypochondriacal safety behavior (QSBH) pre COVID-19 was retrospectively assessed. The shortened version only consists of items which could be assessed at the second time point (during COVID-19). ^d^ Preventive behavior pre COVID-19 was retrospectively assessed.

**Table 2:** Results of the dependent (paired) sample t-tests of reassurance, avoidance, and preventive behavior in relation to SARS-CoV-2 and in relation to other diseases in the subgroups female and male, and the age subgroups “young” (18–29), “middle” (30–59), and “old” (≥60 years).

	Reassurance Behavior									Avoidance Behavior									Preventive Behavior								
	SARS-CoV-2		Other Diseases							SARS-CoV-2		Other Diseases							SARS-CoV-2		Other Viruses						
	*M*	*SD*	*M*	*SD*	BCa 99% CI	|*t*|	*df*	*p*	*d*	*M*	*SD*	*M*	*SD*	*BCa 99% CI*	|*t*|	*df*	*p*	*d*	*M*	*SD*	*M*	*SD*	*BCa 99% CI*	|*t*|	*df*	*p*	*d*
Female	6.63	3.75	6.00	3.52	[0.33, 0.91]	5.56	694	<0.001	0.17	4.45	4.21	4.10	4.38	[0.03, 0.67]	2.76	694	0.01	0.08	15.03	3.29	9.36	3.42	[5.33, 6.01]	42.20	694	<0.001	1.69
Male	6.22	3.75	5.53	3.54	[0.06, 1.35]	2.99	187	0.003	0.19	3.96	4.12	4.43	4.87	[−1.02, 0.24]	1.68	187	0.10	0.10	13.77	4.03	8.86	3.51	[4.18, 5.60]	18.33	187	<0.001	1.29
Young	6.15	3.57	5.53	3.48	[0.23, 1.01]	4.09	404	<0.001	0.18	4.28	4.21	3.07	3.88	[0.76, 1.66]	6.91	404	<0.001	0.30	14.63	3.33	8.98	3.49	[5.20, 6.10]	33.55	404	<0.001	1.66
Middle	6.91	3.91	6.24	3.64	[0.22, 1.11]	4.03	344	<0.001	0.18	4.59	4.44	4.99	4.95	[−0.85, 0.08]	2.22	344	0.03	0.08	14.64	3.75	9.54	3.56	[4.59, 5.61]	24.79	344	<0.001	1.40
Old	6.72	3.78	6.11	3.29	[0.01, 1.26]	2.52	136	0.01	0.17	3.99	3.45	5.34	4.22	[−2.01, −0.72]	5.48	136	<0.001	0.34	15.36	3.31	9.28	2.93	[5.31, 6.82]	20.87	136	<0.001	1.94

**Table 3:** Results of the conditional (including time-varying health anxiety [SHAI] and time-invariant intolerance of uncertainty [IU-18] and distress intolerance [Distress intolerance scale]) growth model of SARS-CoV-2 related anxiety.

	Parameter	Estimate	*SE*	*p*
*Fixed effects*				
Initial level	*b*_0_	1.93	0.62	0.002
Time ^a^	*b*_1_	18.33	0.91	<0.001
Time ^2 a^	*b*_2_	−2.96	0.29	<0.001
Health anxiety ^b^	*b*_3_	−0.07	0.09	0.46
Health anxiety ^b^ × Time ^a^	*b*_4_	1.10	0.14	<0.001
Health anxiety ^b^ × Time^2 a^	*b*_5_	−0.18	0.04	<0.001
Intolerance of uncertainty	*b*_6_	−0.01	0.06	0.83
Intolerance of uncertainty × Time ^a^	*b*_7_	0.02	0.09	0.85
Intolerance of uncertainty × Time^2 a^	*b*_8_	0.01	0.03	0.68
Distress intolerance	*b*_9_	0.04	0.09	0.64
Distress intolerance × Time ^a^	*b*_10_	−0.06	0.14	0.68
Distress intolerance × Time ^2 a^	*b*_11_	0.01	0.04	0.77
*Random effects*				
Variance initial level	*b*_0i_	38.60	9.65	<0.001
Variance quadratic slope (Time ^2 a^)	*b*_1i_	1.51	0.39	<0.001
Covariance initial level−quadratic slope (Time ^2 a^)		7.58		
*Goodness-of-fit*				
Akaike information criterion	AIC	30,988.22		
Bayesian information criterion	BIC	31,087.01		

SHAI = Short Health Anxiety Inventory. IU-18 = Intolerance of Uncertainty Scale—short version. An autoregressive covariance structure with heterogeneous variances was selected. ^a^ Time is coded 0–1-2–3, Time^2^ represents the quadratic slope of Time; all time points were retrospectively measured. ^b^ Health anxiety was included as a time-varying covariate with two points of measurement (pre COVID-19 = until January 2020; during COVID-19 = since January 2020). Equation for the conditional growth model: *Y*_ti_ = *b*_0_ + *b*_1_(TIME_ti_) + *b*_2_(TIME_ti_ × TIME_ti_) + *b*_3_(HA(healthanxiety)_ti_ − HA_gmean_) + *b*_4_(HA_ti_ − HA_gmean_)(TIME_ti_) + *b*_5_(HA_ti_ − HA_gmean_)(TIME_ti_ × TIME_ti_) + *b*_6_(IUS_i_ − IUS_gmean_) + *b*_7_(IUS_i_ − IUS_gmean_)(TIME_ti_) + *b*_8_(IUS_i_ − IUS_gmean_)(TIME_ti_ × TIME_ti_) + *b*_9_(DI_i_ − DI_gmean_) + *b*_10_(DI_i_ − DI_gmean_)(TIME_ti_) + *b*_11_(DI_i_ − DI_gmean_)(TIM_ti_ × TIME_ti_) + *b*_0i_ + *b*_1i_ (TIME_ti_ × TIME_ti_) + ε_ti_.

**Table 4:** Results of the moderator analyses on behavior during COVID-19 with health anxiety (SHAI) pre COVID-19 (retrospectively assessed) as predictor and intolerance of uncertainty [IU-18] and distress intolerance [Distress intolerance scale] as moderators.

	Reassurance Behavior								Avoidance Behavior								Preventive Behavior							
	SARS-CoV-2				Other Diseases				SARS-CoV-2				Other Diseases				SARS-CoV-2				Other Diseases			
	*b*	99% CI [LL, UL]		*p*	*b*	99% CI [LL, UL]		*p*	*b*	99% CI [LL, UL]		*p*	*b*	99% CI [LL, UL]		*p*	*b*	99% CI [LL, UL]		*p*	*b*	99% CI [LL, UL]		*p*
HA pre	−0.001	−0.05	0.05	0.96	0.004	−0.03	0.04	0.80	0.004	−0.05	0.06	0.85	0.02	−0.02	0.06	0.24	0.06	0.01	0.11	0.005	0.02	−0.02	0.05	0.30
DI ^a^	0.04	−0.01	0.08	0.03	0.03	0.01	0.06	0.003	0.07	0.02	0.12	0.001	0.04	0.002	0.08	0.01	0.02	−0.03	0.06	0.31	0.03	−0.01	0.06	0.11
IU ^b^	0.01	−0.01	0.04	0.23	−0.004	−0.02	0.01	0.55	0.004	−0.03	0.04	0.74	−0.005	−0.03	0.02	0.55	0.02	−0.01	0.04	0.12	0.01	−0.02	0.03	0.36
HA pre x DI ^a^	−0.003	−0.01	0.004	0.32	−0.001	−0.01	0.004	0.61	0.001	−0.01	0.01	0.76	<0.001	−0.005	0.004	0.98	−0.004	−0.01	0.002	0.11	0.001	−0.01	0.01	0.80
HA pre x IU ^b^	0.003	−0.001	0.01	0.09	0.001	−0.001	0.004	0.18	−0.002	−0.01	0.003	0.29	<0.001	−0.003	0.003	0.89	0.001	−0.003	0.004	0.74	<0.001	-0.004	0.003	0.79
Behavior pre ^c^	0.59	0.49	0.69	<0.001	0.82	0.75	0.89	<0.001	0.50	0.42	0.59	<0.001	0.88	0.82	0.93	<0.001	0.40	0.32	0.49	<0.001	0.72	0.63	0.81	<0.001
*R* ^2^	0.35				0.68				0.34				0.76				0.18				0.46			

SHAI = Short Health Anxiety Inventory. IU-18 = Intolerance of Uncertainty Scale—short version. ^a^ Distress intolerance (measured with the Distress intolerance scale). ^b^ Intolerance of Uncertainty (measured with the IU-18). ^c^ Corresponding behavior pre COVID-19 (retrospectively assessed).

**Table 5:** Results of the moderator analyses on behavior during COVID-19 with health anxiety (SHAI) during COVID-19 as predictor and intolerance of uncertainty [IU-18] and distress intolerance [Distress intolerance scale] as moderators.

	Reassurance Behavior								Avoidance Behavior								Preventive Behavior							
	SARS-CoV-2				Other Diseases				SARS-CoV-2				Other Diseases				SARS-CoV-2				Other Diseases			
	*b*	99% CI [LL, UL]		*p*	*b*	99% CI [LL, UL]		*p*	*b*	99% CI [LL, UL]		*p*	*b*	99% CI [LL, UL]		*p*	*b*	99% CI [LL, UL]		*p*	*b*	99% CI [LL, UL]		*p*
HA during	0.14	0.10	0.19	<0.001	0.06	0.03	0.09	<0.001	0.002	−0.05	0.05	0.94	0.03	−0.01	0.06	0.08	0.15	0.11	0.20	<0.001	0.05	0.01	0.09	0.002
DI ^a^	0.003	−0.03	0.04	0.81	0.02	−0.004	0.05	0.04	0.07	0.02	0.12	0.001	0.03	0.002	0.07	0.01	−0.01	-0.05	0.04	0.65	0.01	−0.02	0.05	0.30
IU ^b^	−0.01	−0.04	0.01	0.28	-0.01	−0.03	0.005	0.07	0.004	−0.03	0.04	0.79	−0.01	−0.03	0.01	0.41	−0.004	-0.03	0.02	0.69	0.001	−0.02	0.03	0.88
HA during x DI ^a^	0.001	−0.004	0.01	0.72	0.001	−0.004	0.005	0.66	0.001	−0.01	0.01	0.70	0.001	−0.004	0.01	0.51	−0.002	-0.01	0.003	0.43	0.002	−0.004	0.01	0.51
HA during x IU ^b^	0.001	−0.003	0.004	0.65	0.001	−0.002	0.003	0.63	−0.001	−0.005	0.003	0.63	<0.001	−0.003	0.003	0.96	−0.001	-0.005	0.002	0.30	−0.001	−0.004	0.003	0.61
Behavior pre ^c^	0.47	0.37	0.57	<0.001	0.77	0.70	0.85	<0.001	0.50	0.42	0.59	<0.001	0.87	0.81	0.93	<0.001	0.36	0.28	0.45	<0.001	0.70	0.62	0.79	<0.001
*R^2^*	0.42				0.70				0.34				0.76				0.25				0.47			

SHAI = Short Health Anxiety Inventory. IU-18 = Intolerance of Uncertainty Scale—short version. ^a^ Distress intolerance (measured with the Distress intolerance scale). ^b^ Intolerance of Uncertainty (measured with the IU-18). ^c^ Corresponding behavior pre COVID-19 (retrospectively assessed).

## Supplementary Materials

The following are available online at https://www.mdpi.com/1660-4601/17/19/7241/s1, Table S1: Results of the correlational analyses on age (1), sex (2), education (3), profession (4; students vs. others), physical disease (6), immunosuppressive medication (7), mental disorder (8), health anxiety (SHAI) pre (9) and during COVID-19 (10), reassurance, avoidance and preventive behavior during COVID-19 (11–16), and anxiety in relation to SARS-CoV-2 (17–20) and to other diseases (21–23).
