# Supplementary material for: Emotional and Behavioral Consequences of the COVID-19 Pandemic: The Role of Health Anxiety, Intolerance of Uncertainty, and Distress (In)Tolerance
Source: Int J Environ Res Public Health. 2020 Oct 3;17(19):7241. doi: 10.3390/ijerph17197241 (PMC7578938; doi:10.3390/ijerph17197241)
Supplement: Supplementary file 1 [file ijerph-17-07241-s001.pdf]

Supplementary material

Table S1. Results of the correlational analyses on age (1), sex (2), education (3), profession (4; students vs. others), physical disease (6), immunosuppressive medication (7), mental disorder (8), health anxiety (SHAI) pre (9) and during COVID-19 (10), reassurance, avoidance, and preventive behavior during COVID-19 (11–16), and anxiety in relation to SARS-CoV-2 (17–20) and to other diseases (21–23).

		1	2	3	4	5	6	7	8	9	10	11	12	13	14	15	16	17	18	19	20	21	22	23
1	Age	1																						
2	Sex	0.13**	1																					
3	Education [a]	0.07 *	0.10*	1																				
4	Profession: student vs. others	−0.72**	0.15**	0.49**	1																			
5	Physical disease [b]	0.25**	0.05	0.22**	−0.18**	1																		
6	Medication [b]	0.10**	0.02	0.17**	−0.13**	0.30**	1																	
7	Mental disorder [b]	−0.02	0.11 *	0.11*	−0.03	0.07*	−0.002	1																
8	HA pre	−0.09**	0.34 *	−0.001	0.30	0.37**	0.37*	0.48**	1															
9	HA during	−0.06	0.29	−0.05	0.33	0.35*	0.41**	0.49**	0.79**	1														
10	Reassurance SARS-CoV-2 [c]	0.10**	0.12	−0.06	0.19	0.17	0.16	0.19*	0.31**	0.48**	1													
11	Reassurance disease [c]	0.10**	0.16	−0.13**	0.20*	0.18	0.23*	0.24**	0.43**	0.49**	0.61**	1												
12	Avoidance SARS-CoV-2 [c]	−0.01	0.18*	−0.05	0.13	0.15	0.18	0.22*	0.16**	0.20**	0.12**	0.22**	1											
13	Avoidance disease [c]	0.23**	0.15	−0.07	0.25**	0.17	0.14	0.22*	0.19**	0.27**	0.30**	0.35**	0.71**	1										
14	Preventive SARS-CoV-2 [c]	0.08 *	0.21**	−0.09*	0.18*	0.15	0.15	0.12	0.18**	0.36**	0.48**	0.32**	0.05	0.20**	1									
15	Preventive disease [c]	0.06	0.13	−0.11**	0.15	0.19*	0.18*	0.12	0.16**	0.24**	0.36**	0.42**	0.14**	0.21**	0.45**	1								
16	Anxiety SARS-Cov-2 Dec [d]	0.01	0.20	−0.11**	0.21	0.32*	0.41**	0.23	0.12**	0.20**	0.17**	0.17**	0.04	0.10**	0.19**	0.15**	1							
17	Anxiety SARS-CoV-2 Jan [d]	−0.03	0.29	−0.10**	0.30	0.32	0.40**	0.29	0.17**	0.26**	0.21**	0.21**	0.06	0.10**	0.19**	0.20**	0.65**	1						
18	Anxiety SARS-CoV-2 Mar [d]	−0.12**	0.29	−0.08*	0.39*	0.34	0.38*	0.37*	0.34**	0.53**	0.38**	0.27**	0.10**	0.14**	0.39**	0.16**	0.29**	0.41**	1					
19	Anxiety SARS-CoV-2 week [d]	−0.06	0.30	−0.03	0.31	0.33	0.37*	0.40*	0.31**	0.54**	0.43**	0.30**	0.06	0.14**	0.42**	0.24**	0.29**	0.39**	0.76**	1				
20	Anxiety disease Dec [d]	0.05	0.30	−0.04	0.30	0.37*	0.40**	0.39**	0.44**	0.40**	0.24**	0.31**	0.11**	0.18**	0.16**	0.15**	0.34**	0.38**	0.29**	0.31**	1			
21	Anxiety disease Jan [d]	0.06	0.36*	−0.06	0.30	0.37*	0.43**	0.42**	0.45**	0.42**	0.23**	0.32**	0.11**	0.20**	0.16**	0.16**	0.33**	0.40**	0.32**	0.35**	0.91**	1		
22	Anxiety disease Mar [d]	0.07	0.30	−0.03	0.29	0.35*	0.35*	0.37*	0.46**	0.45**	0.26**	0.34**	0.13**	0.22**	0.18**	0.18**	0.31**	0.38**	0.34**	0.35**	0.89**	0.89**	1	
23	Anxiety disease week [d]	0.08*	0.33	−0.03	0.27	0.38*	0.38*	0.38*	0.45**	0.45**	0.26**	0.35**	0.14**	0.17**	0.18**	0.21**	0.31**	0.38**	0.30**	0.35**	0.85**	0.88**	0.92**	1

Profession, physical disease, medication and mental disorder were treated as dichotomous variables, sex as a categorial variable, education, health anxiety, all behavior-related variables and anxiety in relation to SARS-CoV-2 and to other diseases as ordinal variables and age as a metric variable. Phi Coefficients were calculated for correlations between dichotomous variables, Cramer's v for correlations between dichotomous and categorial variables, Spearman's correlations for correlations between ordinal or ordinal and metric variables and point-biserial correlations for correlations between metric and dichotomous/categorial variables. SHAI = Short Health Anxiety Inventory. [a] Education: 0 = no formal education, 1 = primary school or basic school leaving qualification, 2 = secondary school certificate, 3 = advanced technical college certificate, 4 = higher education entrance qualification, 5 = university degree. [b] 1 = yes, 0 = no. [c] during COVID-19 behavior. [d] Anxiety related to SARS-CoV-2 and other severe diseases in December (Dec) 2019, January (Jan) 2020 and March (Mar) 2020 were retrospectively assessed.

** $p < 0.01$, * $p < 0.05$
